# Remote Subcutaneous Needling to Suppress the Irritability of Myofascial Trigger Spots: An Experimental Study in Rabbits

**DOI:** 10.1155/2012/353916

**Published:** 2012-12-25

**Authors:** Zhonghua Fu, Yueh-Ling Hsieh, Chang-Zern Hong, Mu-Jung Kao, Jaung-Geng Lin, Li-Wei Chou

**Affiliations:** ^1^FSN Institute, Nanjing University of Chinese Medicine, Nanjing 210029, China; ^2^Department of Physical Therapy and Graduate Institute of Rehabilitation Science, China Medical University, Taichung 40402, Taiwan; ^3^Department of Physical Therapy, Hungkuang University, Taichung 43302, Taiwan; ^4^Department of Physical Medicine and Rehabilitation, Yangming Branch, Taipei City Hospital, Taipei 11146, Taiwan; ^5^Department of Physical Therapy and Assistive Technology, National Yang-Ming University, Taipei 11221, Taiwan; ^6^School of Chinese Medicine, College of Chinese Medicine, China Medical University, Taichung 40402, Taiwan; ^7^Department of Physical Medicine and Rehabilitation, China Medical University Hospital, 2 Yuh-Der Road, Taichung 40447, Taiwan

## Abstract

*Objective*. To obtain electrophysiological effects of Fu's subcutaneous needling (FSN) on needling distance by assessment of endplate noise (EPN) recorded from the myofascial trigger spots (MTrSs) in rabbit skeletal muscle. *Method*. Eighteen New Zealand rabbits weighing 2.5–3.0 kg were randomly divided into two groups as follows: proximal needling (PN) group and distal needling (DN) group. The needling procedure followed the instructions described by the inventor of FSN, including needling insertion and swaying movement. The amplitudes of EPN on the MTrS region of BF muscle were recorded as an index of MTrS irritability. Random sampling of EPN tracings were taken for further analyses before, during, and after FSN treatment. *Results*. In PN and DN groups, the trends of EPN amplitude alterations were similar at conditions before, during, and after FSN treatment. The degree of reduction in the EPN amplitude in PN group was significantly higher than that in DN group. There were no significant changes in EPN amplitudes in the MTrS of contralateral BF without FSN intervention either in DN or PN group. *Conclusion*. The irritability of proximal MTrSs could be modulated after ipsilateral FSNs. The placement of FSN may affect the effectiveness of suppression of irritability of MTrSs.

## 1. Introduction

Myofascial trigger point (MTrP) is the most tender (hyperirritable) spot in a taut band of skeletal muscle fibers, characterized by a specific pattern of referred pain and local twitch responses (LTR) [1, 2]. Based on the studies on both human and animal subjects, it has been demonstrated that there are multiple sensitive loci in an MTrP region [3, 4]. These sensitive loci are probably nociceptors located in the endplate zone [5]. The prevalence of endplate noise (EPN), as recorded by an electromyographic (EMG) equipment, is significantly higher in an MTrP region than in a non-MTrP region [6, 7] and is highly correlated with the irritability (sensitivity) of an MTrP [8]. Recently, it has also been found that the changes in EPN amplitude significantly correlated with the changes in MTrP irritability [9, 10]. Therefore, MTrP irritability can be objectively assessed with the prevalence or amplitude changes of EPN that are recorded in the MTrP region. The advantage of the amplitude changes of EPN could be the real time recording the alternations of MTrP irritability, that the prevalence of EPN could not obtain. An animal model of MTrP has been established since 1994 [3] for various studies [11–15]. The rabbit myofascial trigger spot (MTrS) is similar to human MTrP in many aspects. EPN can also be recorded from MTrS and can be used for the assessment of therapeutic effectiveness of various modalities [16–18].

Needling therapy includes any treatment with one or more needles. The needle used for medical treatment can be purely a solid metal rod with a sharp tip such as traditional acupuncture needle or can be specifically made with a central hollow as a pathway of drug for injection such as a regular injection needle used in general medical practice. “Dry needling” is a pure penetration of a needle through the skin without introduction of any drug. For dry needling, the site of treatment can be at the site of pain (direct dry needling) or far from the site of pain (remote dry needling). Either direct dry needling or remote dry needling, the following techniques can be used: traditional acupuncture [19–27], dry needling with multiple rapid insertions [9, 10, 28–34], and dry needling with electrical stimulation (similar to electrical acupuncture) [35–37]. However, superficial dry needling [38, 39] and deep dry needling for soft tissue release are usually performed directly over the painful lesions only, and Fu's subcutaneous needling [40–42] is usually applied over the remote non-painful site only.

**Figure 1:**
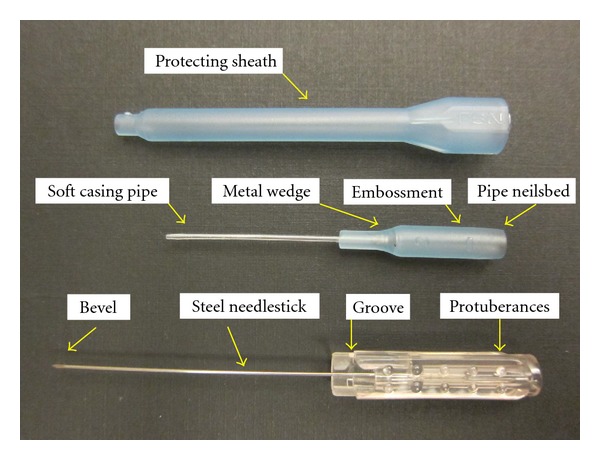
Three parts of Fu's subcutaneous needling.

**Figure 2:**
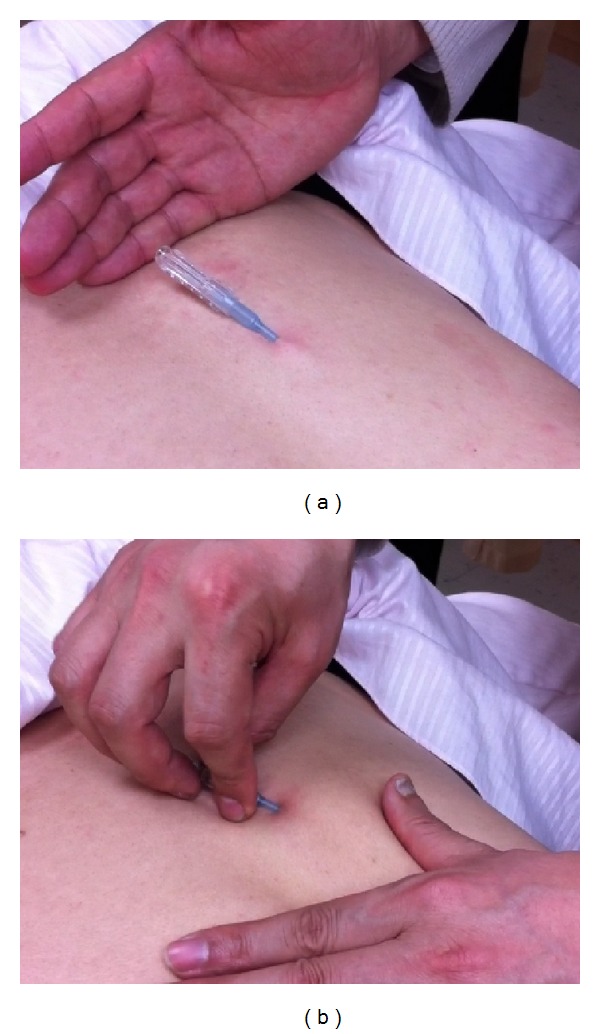
(a) The Fu's subcutaneous needling (FSN) is placed to the subcutaneous tissue, and (b) swayed during treatment.

Fu's subcutaneous needling (FSN), one type of remote subcutaneous needling, is a therapeutic approach mainly for musculoskeletal painful disorders. This procedure is performed by inserting a special trocar needle (Figure 1) into the subcutaneous layer around the afflicted spot to achieve the desired effect. In English, it was first described in the article by Fu and Xu in the journal of “The Pain Clinic” [40]. Since then, a series of research papers have been reported and claimed the clinical effectiveness, including painful diseases [43], lower back pain [41], and MTrP in the neck [42]. The way of manipulation of FSN is different from traditional acupuncture and other needling approaches. The FSN is inserted into non-diseased areas based on the nature of trigger points and the needle placement is restricted to the subcutaneous tissue (Figure 2(a)); the tip of FSN is directed to the painful region. Specifically, FSN should be swayed from side to side (Figure 2(b)) and may be retained in the subcutaneous tissues for a prolonged period of time to obtain a curative effect [40, 44].

Although many clinical evidences of FSN have been reported as mentioned above, its underlying mechanism is still unclear and needs to be further investigated by using the animal study. In this study, we aim to obtain electrophysiological confirmation of the remote effect of FSN and compare the effectiveness of needling sites with different distance from the painful site based on the assessment of EPN recorded from the MTrSs in rabbit skeletal muscle.

## 2. Materials and Methods

### 2.1. General Design

**Figure 3:**
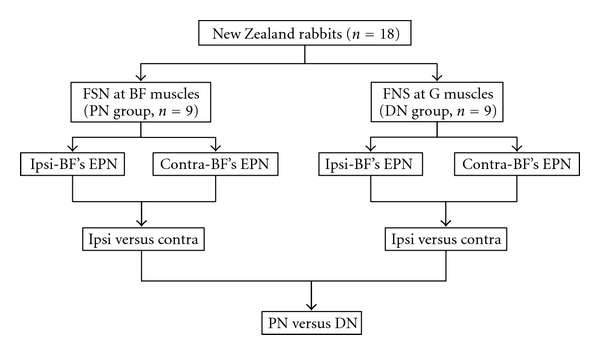
Flow chart for the animal study. (BF = bicep femoris; Contra = EPN recording side contralaterally to FSN side; DN = distal needling; EPN = endplate noise; FSN = Fu's subcutaneous needling; G = gastrocnemius; Ipsi = EPN recording side ipsilaterally to FSN side; PN = proximal needling).

**Figure 4:**
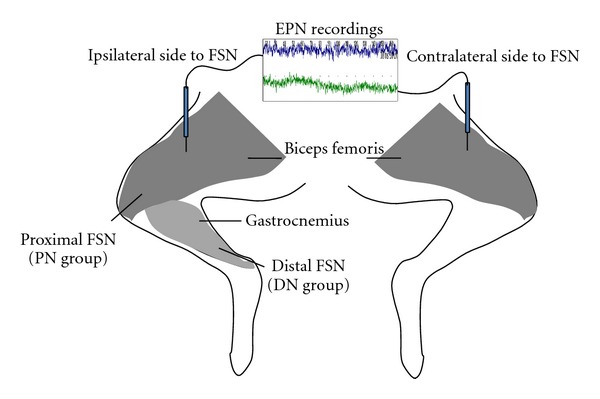
The sites and sides of endplate noise (EPN) recordings, and that for Fu's subcutaneous needling (FSN) for animals in proximal needling (PN) and distal needling (DN) groups.

**Figure 5:**
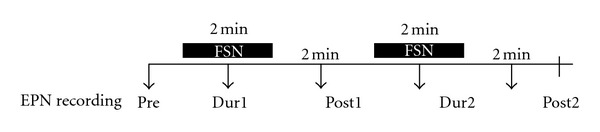
Sequences of Fu's subcutaneous needling (FSN) and endplate noise (EPN) assessment in the whole course of the experiment.

The FSN-induced EPN alterations on the MTrS irritability (assessed with EPN amplitude changes in the electromyographic recordings) at bilateral biceps femoris (BF) muscle were examined. A total of 18 rabbits were randomly divided into 2 groups based on the placement of FSN (Figure 3): on subcutaneous layer over the insertion region of the BF muscle (proximal needling, PN group, *n* = 9, Figure 4) and on subcutaneous layer over the insertion region of the gastrocnemius muscle (distal needling, DN group, *n* = 9, Figure 4). The FSN treatment side of animal's hindlimb was randomly selected by manipulators. FSN treatments were repeated for two times (Dur1 and Dur2) with an interval of two minutes between the two needling therapies. Continuous tracings of EPN were recorded from BF muscle before (Pre-), during the 1st needling (Dur1-), after the 1st needling (Post1-), during the 2nd needling (Dur2-), and after the 2nd needling (Post2-) of FSN in anesthetized animals (Figure 5).

The changes in MTrS irritability (EPN amplitude) in PN and DN groups were compared for all EPN recoding periods including Pre-, Dur1-, Post1-, Dur2-, and Post2-FSN conditions.

### 2.2. Animals Care and Preparation

The experiments were performed on adult New Zealand rabbits (body weight 2.5–3.0 kg). Each animal was housed individually in a standard polycarbonate tub cage lined with wood chip beddings and had free access to food and water. The cage was placed in an air-conditioned room (25 ± 1°C), with noise level less than 40 dBA and a 12-hour alternating light-dark cycle (6 : 00 am to 6 : 00 pm). The ethical guidelines of the International Association for Study of Pain in animals were followed [45]. All animal experiments were conducted with procedures approved by the Animal Care and Use Committee of a university in accordance with the Guidelines for Animal Experimentation.

Before anesthesia, the most tender spots (i.e., MTrS) of BF muscle were identified by finger pinch. The animal's reactions to the pinch stimulation were observed (withdrawal of the lower limb, turning its head, screaming, etc.) to confirm the exact location of an MTrS. The painful region was marked on the skin with an indelible marker for electrophysiological assessment. Then the animals were anesthetized with 2% isoflurane (AErrane, Baxter Healthcare of Puerto Rico, PR, USA) in oxygen flow for induction followed by a 0.5% maintenance dose [46]. Body temperature, monitored by the thermistor probe of a thermometer (Physiotemp Instrument, Clifton, NJ, USA) in the rectum, was maintained at approximately 37.5°C using a body temperature control system consisting of thermostatically regulated DC current heating pad and an infrared lamp. The hindlimbs of anesthetized rabbits were shaved and cleaned with povidone-iodine solution. The skin of the lateral thigh in one randomly selected side was incised to expose the BF muscle, which were served as an EPN recording site. The marked spots in the BF muscle were grasped between two fingers from behind the muscle and the muscle is palpated by gently rubbing (rolling) it between the fingers to find a taut band. A taut band feels like a clearly delineated “rope” of muscle fibers and is roughly 2-3 mm or more in diameter. The fibers of the taut band are unmistakably firmer in consistency than the surrounding muscle.

### 2.3. Fu's Subcutaneous Needling Manipulation

The FSN manipulation procedure was followed the instructions described by the inventor of FSN, including needling insertion and swaying movement [40, 41]. Step 1: quickly penetrate the needle obliquely through the skin. The angle between the needle and skin is about 20°–30°. Make sure the needle tip is not too deep and stop when the needle tip just touches the muscle layer. Step 2: draw back the needle a little to the subcutaneous layer. Step 3: push forward the needle parallel to the skin surface (maintaining in the subcutaneous layer) until the whole soft tube is under the skin and leave the needle in the subcutaneous layer. Step 4: draw the steel needle back 3 millimeters to make the steel tip wrapped in the soft tube, then sway the needle smoothly and rhythmically from one side to another horizontally at a rate about 200 times in 2 minutes. During the swaying movement, the needle remained in the subcutaneous layer (Figure 2). The same investigator performed all needling procedures for all rabbits.

### 2.4. Recording of Endplate Noise (EPN)

#### 2.4.1. Electromyography Setting

For EPN assessment, a two-channel digital EMG machine (Neuro-EMG-Micro, Neurosoft, Ivanovo, Russia) and monopolar needle electrodes (37-mm, disposable, Teflon-coated, model 902-DMF37-TP; VIASYS/Cardinal Healthcare, Dublin, OH, USA) were used. The gain was set at 20 *μ*V per division for recordings from both channels. Low-cut frequency filter was set at 100 Hz and the high-cut at 1,000 Hz. Sweep speed was 10 ms per division. The search needle for EPN recording was inserted into the MTrS region and was connected to the 1st channel of the EMG machine. The control needle was inserted into the non-taut band region near the MTrS in the same muscle and was connected to the 2nd channel. A common reference needle electrode for each channel was placed on the incised skin and connected to both channels via a y-connector.

#### 2.4.2. Search for Endplate Noise

An investigator who was blind to the group assignment performed this procedure. The search needle was inserted into the MTrS region in a direction parallel to the muscle fibers at an angle of approximately 60° to the surface of the muscle. After initial insertion just short of the depth of the MTrS or to comparable depth in the case of control sites, the needle was advanced very slowly. Each advance was of minimal distance (about 1 mm). When the needle approached an active locus (EPN locus), the continuous distant electrical activity, that is, EPN, can be heard. A site was an active locus when EPN was identified if (1) EPN-like potentials persisted continuously for more than 300 msec, (2) the potentials had an amplitude of >10 *μ*V (which was more than twice the instrumentation noise level of 4 *μ*V that was observed in control recordings taken at the beginning and at completion of each track), and (3) the adjacent control channel was not recording potentials greater than the instrumentation noise level. As soon as the EMG activity (EPN) with an amplitude higher than 10 *μ*V could be recorded, the examiner stopped to move and remain the needle there to ensure that this EPN could run continuously on the recording screen with constant amplitudes. Then, continuous EPN tracing was recorded throughout the whole course of FSN treatment and provided the opportunity for continuous visual observation of EPN changes on the EMG screen. The whole EPN tracing found in MTrS of BF muscle were recorded for the analysis of amplitude changes.

#### 2.4.3. Measurement of the Amplitude of Endplate Noise

Randomly selected five samples of EPN recordings (50 msec for each) were taken before (Pre-), during (DN-) and after (Post-) the FNS treatment for both groups. The mean amplitude of EPN was analyzed and calculated using embedded software in the Neuro-EMG-Micro equipment.

### 2.5. Date Analysis

Data were expressed as the mean ± standard error of the mean (SEM). The serial differences in EPN amplitude among Pre-, Dur-, and Post-FSN conditions in each group were carried out using an ANOVA followed by a Scheffe's post-hoc analysis. The alteration of EPN amplitude was calculated as follow: percentage of EPN alteration = ((data in Dur- or Post-FNS condition − data in Pre-FSN condition)/data in Pre-FSN condition)/100%. The differences of reduction percentage between EPN recording sides ispi- and contra-laterally to FSN side and between PN and DN groups in each recording side were tested by Student *t*-test. A *P* value of <0.05 was considered to be statistically significant. All data was analyzed using Statistical Package for the Social Sciences (SPSS, version 12.0) for Windows.

## 3. Results

### 3.1. Effects of FSN on Serial EPN Recordings

**Figure 6:**
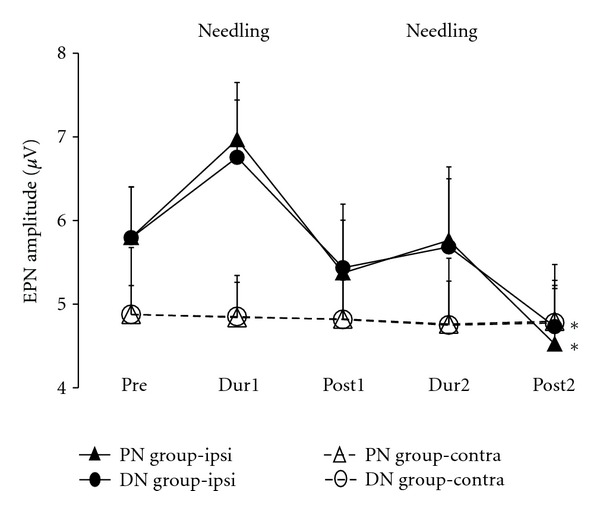
The serial changes of the mean amplitudes of endplate noise (EPN) in groups with proximal (PN group) and distal (DN group) Fu's subcutaneous needling (FSN). ipsi and contra indicate the EPN recording ipsilaterally and contralaterally to FSN. *Indicates *P* < 0.05 tested by ANOVA.

The serial alterations of the mean EPN amplitude of the ipsilateral and contralateral BF muscle in Pre- Dur1-, Post1-, Dur2-, and Post2-FNS conditions in PN and DN groups were demonstrated in Table 1 and Figure 6. In PN group, the mean EPN amplitudes recorded from MTrS of BF muscle ipsilaterally to FSN were significantly different among Pre- Dur1-, Post1-, Dur2-, and Post2-FNS conditions (*P* = 0.001). The significant reduction of EPN amplitudes was found in Post2-FSN (*P* = 0.001) condition when compared with those in Dur1-FSN condition. But the EPN amplitudes recorded from MTrS of BF muscle contralaterally to FSN were no significant differences among Pre- Dur1-, Post1-, Dur2-, and Post2-FNS conditions (*P* > 0.05).

The trend of EPN amplitude in DN group was similar to those in PN group regardless of EPN recording sides. There were significant differences among Pre-, Dur1-, Post1-, Dur2-, and Post2-FSN conditions in the DN group in ipsilateral EPN recording side (*P* = 0.006), but not found in contralateral recording side (*P* > 0.05). The significant reduction of EPN amplitudes in ipsilateral recording side was also found in Post2-FSN (*P* = 0.008) condition when compared with those in Dur1-FSN condition in DN group.

**Figure 7:**
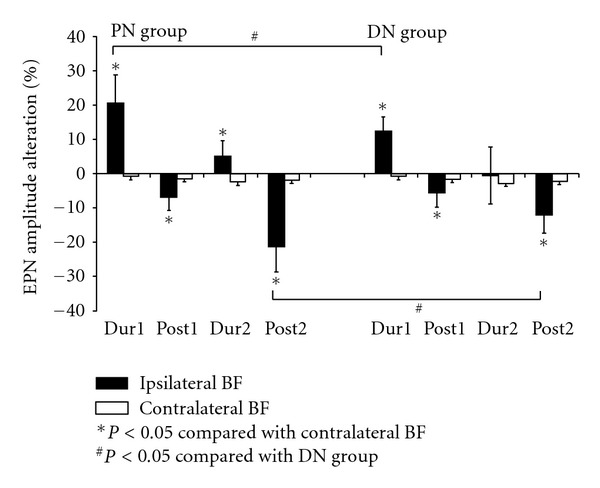
The percentages of EPN amplitude alteration in bilateral biceps femoris (BF) muscle in each condition when compared with the data before proximal and distal manipulation of Fu's subcutaneous needling (PN and DN group). Abbreviations: BF = biceps femoris; DN = distal needling; EPN = endplate noise; PN = proximal needling.

In the PN group, the percentages of EPN amplitude alteration of ipsialteral BF were significantly increased in Dur1- and Dur2-FSN levels, and then was significantly decreased to much lower level in Post1- and Post2-FSN levels when comparing with those data recorded from contralateral BF. In the DN group, the trend of percentage of EPN alteration was similar (Figure 7).

### 3.2. Distance Effect of Fu's Subcutaneous Needling

After the whole course of treatments, the percentages of EPN amplitude alterations reduced significantly (*P* < 0.05) in the ipsilateral PN and DN groups. There were significant differences on the amount of reduction between PN and DN groups. The percentages of amplitude alterations were significantly reduced in the PN group when compared with those in the DN groups (Figure 6).

## 4. Discussion

### 4.1. Summary of Important Findings in This Study

To our knowledge, the present study is the first animal study to investigate the neural mechanism of the remote effects of FSN. In this study, we have found that the irritability of proximal MTrS at BF muscle could be modulated by the remote effect of FSN at ipsilateral distant leg. The placement of FSN (distance to the MTrS) may be a crucial factor on the suppressive effectiveness of irritability of MTrS.

### 4.2. Insertion Point and Needling Direction for Fu's Subcutaneous Needling

Fu's Subcutaneous Needling is a therapeutic approach mainly for musculoskeletal painful disorders, including myofascial pain syndrome. The choosing of insertion points and needle direction were based on Fu's experience and some rules in a famous ancient Chinese medical book (*Yellow Emperor's Internal Classic*) for pain syndromes (also called *Bi *syndromes) [41].

Some principles and clinical experiments of the locations and direction of the FSN insertion point(s) have been mentioned as followed: (1) the FSN needle does not reach the pain area, no matter how short the distance between needling site and painful site [40]; (2) the distance should vary with individuals. For pain involving a large-area or for severe pain, the distance should be longer, and the insertion point is chosen near to the painful point if the painful area is small [40]; (3) needling directions are well correlated to the outcome [41].

### 4.3. Difference between Dry Needling (Acupuncture) and Fu's Subcutaneous Needling

Traditional acupuncture therapy is probably the oldest type of dry needling. The manipulation of the acupuncture needle is “fast in and fast out” or “rotation,” but the FSN is moved from one side to another smoothly and rhythmically [41]. In regular acupuncture therapies, immediate pain relief can be obtained if the patient experiences “*De-qi*” reaction during therapy. The mechanism of local effects on the site of needling for the immediate relief of pain after acupuncture or dry needling has been consider to be mediated via the neural pathway [38, 47]. On the contrary, the FSN claims that the tip of the FSN needle had better not reach the painful spots [40], but good effectiveness could be obtained without “*De-qi*” or local twitch response. Fu and his colleagues [41] have hypothesized that no “*De-qi*” elicited during FSN maybe related to the paucity of free nerve endings and proprioceptive receptors in the subcutaneous layer.

### 4.4. Possible Mechanism of Fu's Subcutaneous Needling for Pain Control

The subcutaneous layer contains adipose tissue and connective tissue. Langevin and his colleagues [48] hypothesized that mechanical coupling between the needle and connective tissue with winding of tissue around the needle during needle rotation transmits a mechanical signal to connective tissue cells that may explain local and remote, as well as long-term effects of acupuncture. Fu and Xu [40] presumed that the mechanism of FSN probably acts on the mechanical forces of the subcutaneous layer by regulating the homeostasis of the connective tissue. At the later report, Fu and his colleagues [42] thought that there was little possibility that the nervous system could be involved in the action of FSN.

### 4.5. Possible Mechanism of the Remote Effect of Fu's Subcutaneous Needling

Although many clinical evidences of the remote influences of FSN have been reported [41–43], its underlying mechanism is still unclear and needs to be further investigated by using animal studies. The animal model with MTrS was well established previously [3, 18] and served in this study for further investigating the possible mechanism of remote effect after FSN. MTrP irritability can be objectively assessed with the prevalence or amplitude changes of EPN that are recorded in the MTrP region. It has been found that the changes in EPN amplitude significantly correlated with the changes in MTrP irritability in a recent study [9].

As demonstrated in previous study [15], the irritability of MTrS at BF muscle (proximal MTrS) could be modulated by the remote effect of dry needling ipsilaterally or contralaterally at MTrS of gastrocnemius muscle (distant MTrS). This remote effect depends on an intact afferent pathway from stimulating site to the spinal cord and a normal spinal cord function at the level corresponding to the innervations of the proximally affected muscle. However, the electrophysiological findings in this study demonstrated that FSN to MTrSs of distal muscles of ipsilateral gastrocnemius muscle could initially increase the irritability of MTrS in proximal muscle (BF), followed a suppression effect after cessation of needling, but not found in the contralateral side.

There weren't any contralateral remote effects found in this study. Therefore, it is very likely that the mechanism of FSN is not related to neural mechanism, but due to the effect of mechanical connective tissue reaction as mentioned above. A direct mechanical connective tissue reaction cannot be transmitted to the other side of body. Similarly, the transmitting reaction of mechanical force is much stronger for the short-distance reaction than that of a longer distance. That is probably the reason why we obtained a stronger effect in PN than that in DN group.

### 4.6. Limitation of This Study

The most critical limitation of this study is the difficulty to confirm the correlation between the alterations of EPN amplitude and pain intensity in rabbit MTrS. We can confirm that in human study. Since there are many similarities between rabbit MTrS and human MTrP, we can reasonably assume that the amplitude of EPN recorded from a rabbit MTrS is related to the irritability of the MTrS similar to that in human MTrP. At least, our study could confirm that the FSN effect is not related to the psychological effect that previous author concerned [40]. It is also less likely that FSN effect is related to neural connection.

Lack of follow-up assessments for the long-term remote effect after the cessation of the FSN stimulation is another deficiency of this study. In addition, no placebo group (minimal/sham needling or other acupoint) was used in our study. It is obvious that great attention should be paid to all limiting factors discussed in this study. Further studies on the mechanical force effects based on a long-term follow-up of EPN amplitude after FSN treatments may be helpful for straightening out the deficiency of this study.

## 5. Conclusion

This study confirmed the electrophysiological phenomenon of the remote effect of FSN (one type of remote subcutaneous needling) and the possible pathway in response to this remote effect based on the assessment of EPN recorded for the MTrS in rabbit skeletal muscle which is equivalent to the MTrP in human muscle. It appears that the MTrS irritability can be suppressed after an ipsilateral remote FSN treatment, with a better effectiveness after the proximal FSN therapy than the distal one.

## Figures and Tables

**Table 1:** The serial alterations of EPN amplitudes (*μ*V) in proximal and distal manipulation of Fu's subcutaneous needling (FSN).

Condition	PN group		DN group	
	Ipsi	Contra	Ipsi	Contra
Pre	5.80 ± 0.60	4.88 ± 0.80	5.79 ± 0.61	4.88 ± 0.35
Dur1	6.97 ± 0.47	4.84 ± 0.50	6.76 ± 0.89	4.85 ± 0.41
Post1	5.37 ± 0.63	4.82 ± 0.46	5.44 ± 0.76	4.82 ± 0.64
Dur2	5.76 ± 0.88	4.76 ± 0.79	5.68 ± 0.82	4.75 ± 0.53
Post2	4.52 ± 0.76*	4.80 ± 0.43	4.73 ± 0.74*	4.78 ± 0.41

*P* value^a^	0.001	1.00	0.006	1.00
*poc hoc *test^b^	*Dur1 versus Post2		*Dur1 versus Post2	

^a^Tested by ANOVA.

^b^Tested by Scheffe's method.

*Indicates significant difference (*P* < 0.05) tested by Scheffe's method.

Abbreviations: Contra: EPN recording side contralaterally to FSN side; DN: distal needling; EPN: endplate noise; Ipsi: EPN recording side ipsilaterally to FSN side; PN: proximal needling.
